# The Stability and Repeatability of Spontaneous Sympathetic Baroreflex Sensitivity in Healthy Young Individuals

**DOI:** 10.3389/fnins.2018.00403

**Published:** 2018-06-14

**Authors:** Sarah L. Hissen, Khadigeh El Sayed, Vaughan G. Macefield, Rachael Brown, Chloe E. Taylor

**Affiliations:** ^1^School of Science and Health, Western Sydney University, Sydney, NSW, Australia; ^2^School of Medicine, Western Sydney University, Sydney, NSW, Australia; ^3^Baker Heart and Diabetes Institute, Melbourne, VIC, Australia; ^4^Neuroscience Research Australia, Sydney, NSW, Australia

**Keywords:** repeatability, blood pressure, baroreflex sensitivity, microneurography, muscle sympathetic nerve activity

## Abstract

Spontaneous sympathetic baroreflex sensitivity (BRS) is a valuable tool for assessing how well the baroreflex buffers beat-to-beat changes in blood pressure. However, there has yet to be a study involving appropriate statistical tests to examine the stability of sympathetic BRS within an experimental session and the repeatability between separate sessions. The aim of this study was to use intra-class correlations, ordinary least products regression, and Bland–Altman analyses to examine the stability and repeatability of spontaneous sympathetic BRS assessment. In addition, the influence of recording duration on values of BRS was assessed. In eighty-four healthy young individuals (49 males, 35 females), continuous measurements of blood pressure, heart rate and muscle sympathetic nerve activity (MSNA) were recorded for 10 min. In a subgroup of 13 participants (11 male, 2 female) the measurements were repeated on a separate day. Sympathetic BRS was quantified using MSNA burst incidence (BRS_inc_) and total MSNA (BRS_total_) for the first 5-min period, the second 5-min period, and a 2-min segment taken from the second 5-min period. Intra-class correlation coefficients indicated moderate stability in sympathetic BRS_inc_ and BRS_total_ between the first and second 5-min periods in males (BRS_inc_ *r* = 0.63, BRS_total_ *r* = 0.78) and females (BRS_inc_ *r* = 0.61, BRS_total_ *r* = 0.47) with no proportional bias, but with fixed bias for BRS_inc_ in females. When comparing the first 5-min with the 2-min period (*n* = 76), the intra-class correlation coefficient indicated poor to moderate repeatability in sympathetic BRS_inc_ and BRS_total_ for males (BRS_inc_ *r* = -0.01, BRS_total_ *r* = 0.70) and females (BRS_inc_ *r* = 0.46, BRS_total_ *r* = 0.39). However, Bland–Altman analysis revealed a fixed bias for BRS_total_ in males and proportional bias for BRS_total_ in females, with lower BRS values for 5-min recordings. In the subgroup, intra-class correlations indicated moderate repeatability for measures of BRS_inc_ (9 male, 2 female, *r* = 0.63) and BRS_total_ (6 male, 2 female, *r* = 0.68) assessed using 5-min periods recorded on separate days. However, Bland–Altman analysis indicated proportional bias for BRS_inc_ and fixed bias for BRS_total_. In conclusion, measures of spontaneous sympathetic BRS are moderately stable and repeatable within and between testing sessions in healthy young adults, provided that the same length of recording is used when making comparisons.

## Introduction

Blood pressure is homeostatically maintained at an optimal level to suit a given task or situation, such as changes in posture, exercise, or mental stress (Benarroch, 2008), through barorflex-mediated changes in total peripheral resistance, as determined by the degree of vasoconstriction in systemic arterioles, and cardiac output. The ability of the baroreflex to efficiently buffer beat-to-beat changes in blood pressure is known as baroreflex sensitivity (BRS); it does this by modulating both heart rate (cardiac BRS) and muscle sympathetic nerve activity (MSNA) (sympathetic BRS) (Benarroch, 2008; Wehrwein and Joyner, 2013). Spontaneous sympathetic BRS is typically quantified by binning the diastolic pressures into either 1, 2, or 3 mmHg bins and plotting the mean for each bin against either MSNA burst incidence, burst strength (amplitude/area) or integrated activity (Halliwill, 2000; Kienbaum et al., 2001). The slope of this relationship provides an individual’s baroreflex sensitivity (Sundlof and Wallin, 1978; Kienbaum et al., 2001). The steeper the slope, the more efficient the baroreflex is in correcting for changes in blood pressure (Charkoudian and Wallin, 2014; Taylor et al., 2014). Hart et al. (2010) have previously found that the sympathetic BRS values determined by the spontaneous burst incidence technique (also known as the threshold method) correlate well with those defined by the “gold-standard" modified Oxford method. Thus, the diastolic pressure-MSNA burst incidence slope produced via the spontaneous threshold method has been accepted as a robust non-pharmacological alternative to the modified Oxford method (Hart et al., 2010).

Kienbaum et al. (2001) have theorized that the modulation of muscle sympathetic outflow via the baroreflex, has two central nervous system pathways; one modulating the gating (i.e., incidence) of bursts, and the other modulating the strength (i.e., amplitude) of a burst. It has been reported that computation of sympathetic BRS values is more successful when quantified using the gating (incidence) of sympathetic bursts rather than the strength of the sympathetic outflow (amplitude/area of MSNA burst) (Kienbaum et al., 2001). Keller et al. (2006) later went on to combine the incidence and strength of sympathetic bursts in the quantification of sympathetic BRS. Another method of analysis is the segregated signal averaging approach, developed by Halliwill (2000). This technique involves the quantification of total integrated MSNA across all cardiac cycles which is then averaged for each diastolic pressure bin. These methods of assessing spontaneous sympathetic BRS have been used to evaluate the cardiovascular benefits of lifestyle interventions, such as exercise training (Laterza et al., 2007) and diet (Lambert et al., 2011). They have also been applied to studies of diurnal variation in baroreflex function (Hissen et al., 2015) and the effects of heat stress (Keller et al., 2006). To confidently report enhancements in baroreflex function following an intervention, it is essential to assess spontaneous sympathetic BRS with a technique that is both accurate and repeatable. However, while the accuracy of the spontaneous baroreflex technique is accepted (Hart et al., 2010), a comprehensive assessment of the repeatability of this method has yet to be conducted. Dawson et al. (1997) previously reported that the analytical techniques commonly used to measure spontaneous *cardiac* BRS, such as the sequence method, are repeatable between recording sessions. Whilst there is considerable variability in resting MSNA from person to person, inter-individual differences in MSNA have been shown to be reproducible on the same day (Notay et al., 2016), to months and even years between trials (Sundlof and Wallin, 1977; Fagius and Wallin, 1993; Kimmerly et al., 2004). The use of MSNA burst incidence when quantifying sympathetic BRS has previously been shown to be repeatable, whereas methods involving MSNA burst strength have not (Kienbaum et al., 2001). However, these conclusions were made based on minimal statistical analysis (paired *t*-tests), and so the variability between sympathetic BRS values within each participant has yet to be revealed. By assessing segments of data from the same recording period we can assess the stability of sympathetic BRS within subjects.

Therefore, the aim of this study is to employ appropriate statistical tests to determine whether sympathetic BRS, quantified using spontaneous techniques, is stable during the same recording session in healthy young adults. It is hypothesized that sympathetic BRS is highly stable during a single recording session. It is also hypothesized that recording periods of longer durations are associated with greater diastolic pressure ranges at rest and that poor correlations exist between sympathetic BRS values derived from recording periods of different durations. Therefore, a secondary aim is to determine whether the duration of the recording period influences values of sympathetic BRS. Finally, it is hypothesized that measures of sympathetic BRS are repeatable between experimental sessions on different days. Therefore, the final aim is to assess test–retest repeatability in a subgroup of participants in whom measurements were made on two separate days.

## Materials and Methods

### Participants

This study was a retrospective analysis of unpublished data combined with data presented in previous publications (Hissen et al., 2015; Taylor et al., 2015; El Sayed et al., 2016). The participants were 49 male and 35 female healthy young individuals aged between 18 and 31 years (height 171 ± 10 cm, weight 71 ± 15 kg, BMI 24 ± 5) who did not smoke or take regular medication and had no history of cardiovascular, respiratory, or endocrine disease. From this cohort, 13 participants (2 female) returned to the lab on a separate day (ranging from 3 to 43 months) where the between day test–retest repeatability of sympathetic BRS was examined. Participants were informed of what was involved in the experiment both in writing and verbally before signing a consent form. Each experiment was conducted with the approval of the Human Research Ethics committee, Western Sydney University, and satisfied the Declaration of Helsinki. Participants were instructed to abstain from alcohol and vigorous activity for 24 h before the experiment, and not to consume any caffeine on the day. The changes in hormone levels during the menstrual cycle have been shown to affect MSNA and sympathetic BRS (Minson et al., 2000). Due to this, females were tested in the low hormone (early follicular, days 1–7) phase of their menstrual cycle to minimize the effects of sex hormones on sympathetic BRS.

### Measurements and Experimental Protocol

Participants sat in a semi-recumbent position (45°) while beat-to-beat measurements of ECG, blood pressure, respiration, and MSNA were recorded for 10-min. Heart rate was recorded through an electrocardiogram (0.3–1 kHz) using Ag-AgCl surface electrodes on the chest, sampled at 2 kHz (BioAmplifier, ADInstruments, Sydney, NSW, Australia). Blood pressure was recorded via a finger cuff on the left third or fourth digit (NOVA, Finapres Medical Systems, Netherlands), height-corrected for the distance between the heart and finger, and sampled at 400 Hz. Respiration was measured via a strain gauge transducer (DC-100 Hz; Pneumotrace II, UFI, Morro Bay, CA, United States) wrapped around the chest. This was used to ensure participants were breathing at a normal rate throughout the protocol and were not holding their breath at any point. The common peroneal nerve was mapped out on the leg at the level of the fibular head using external stimulation (constant-current stimuli, 0.2 ms pulses, 2–10 mA) on the surface of the skin at 1 Hz (Stimulus Isolator, ADInstruments, Sydney, NSW, Australia). Once the best site for inserting the microelectrode was located, a tungsten microelectrode (Frederick Haer and Co, Bowdoin, ME, United States) was inserted into the skin and guided to the nerve through weak electrical stimuli (0.02–1 mA) to induce muscle twitches. A reference electrode with 1 mm insulation removed was inserted just under the skin about 1–2 cm from the recording site. Adjustments were made to the microelectrode until it penetrated a muscle fascicle and spontaneous MSNA was apparent. The identity of a muscle fascicle was confirmed through tapping and stretching of the muscle belly to evoke muscle spindle afferent activity, and no increase in afferent activity when stroking the skin (Sundlof and Wallin, 1977). Small adjustments of the microelectrode tip were then taken until spontaneous bursts of MSNA were found. Neural activity was amplified (gain 20,000) and filtered (bandpass 0.3–5.0 kHz) using an isolated headstage (NeuroAmpEx, ADInstruments, Sydney, NSW, Australia), and stored (10 kHz sampling) using a computer-based data acquisition and analysis system (PowerLab 16SP hardware and LabChart 8 software; ADInstruments, Sydney, NSW, Australia). A root-mean-square-processed version of the signal was computed with a moving average of 200 ms. Recording of data did not begin until spontaneous MSNA was achieved and stabilized for at least 10 min.

### Data Analysis

After a stable period of baseline activity (>10 min) had been obtained, a 10-min recording of beat-to-beat values of systolic blood pressure, diastolic blood pressure, mean arterial pressure, R-R interval and MSNA was extracted from LabChart (ADInstruments, Sydney, NSW, Australia). The 10-min recording period was then split into two 5-min periods to determine the stability of spontaneous sympathetic BRS within the same session. A 2-min recording was taken from the second 5-min recording to compare BRS values obtained from recording periods of different duration. The detection and area of each MSNA burst and the quantification of sympathetic BRS was performed using Ensemble (Elucimed Ltd., Wellington, New Zealand). The number of bursts per minute (MSNA burst frequency) and per 100 heartbeats (MSNA burst incidence) was determined for each individual. The following analyses were performed for both of the 5-min periods, and the 2-min recording. In a sub group of 13 participants (11 male, 2 female), a second 5-min recording period was recorded on a separate day to determine test–retest repeatability of spontaneous sympathetic BRS. Two approaches were used for the assessment of sympathetic BRS: the burst incidence method, and a segregated signal averaging approach that incorporates total MSNA.

### Sympathetic Baroreflex Sensitivity: Burst Incidence Method

**FIGURE 1:**
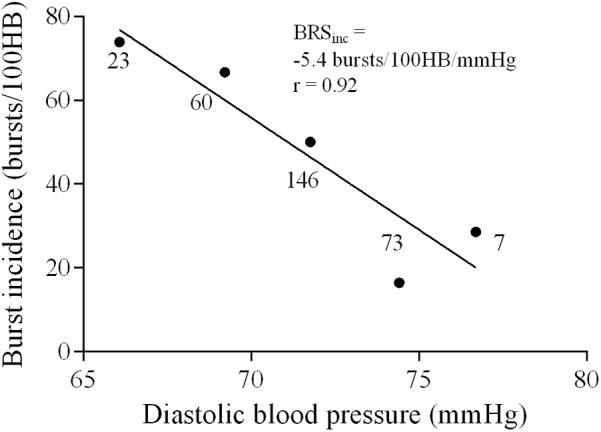
Sympathetic baroreflex assessment in a 24-year-old female using the burst incidence method. MSNA burst incidence is determined for each 3 mmHg diastolic pressure bin and then plotted against diastolic pressure. Number of cardiac cycles per bin indicated next to each data point.

Sympathetic BRS was quantified using methods previously described by Kienbaum et al. (2001). The nerve trace was optimally shifted for each participant for both methods of sympathetic BRS (mean ± SD 1.3 ± 0.1 s) to account for individual variability in sympathetic burst latency. Diastolic pressures were assigned to 3 mmHg bins for each participant to remove potential non-baroreflex stimuli (Ebert and Cowley, 1992; Tzeng et al., 2009). For each bin, the corresponding MSNA burst incidence was determined. Sympathetic BRS was quantified by plotting MSNA burst incidence against the mean diastolic pressure for each bin. Each data point was weighted according to the number of cardiac cycles, as the bins at the highest and lowest diastolic pressures contain fewer cardiac cycles (Kienbaum et al., 2001). The value of the slope, determined via linear regression analysis, provided the sympathetic BRS for the individual, which will be referred to as ‘sympathetic BRS_inc_.’ **Figure 1** illustrates sympathetic BRS_inc_ slope quantified during the first 5-min recording in a 24-year-old female. It is important that the BRS value represents the linear portion fo the baroreflex slope. However, in cases where a sigmoidal relationship was present the weighting procedure limits the influence of saturation and threshold regions, for which there are very few cardiac cycles.

### Sympathetic Baroreflex Sensitivity: Total MSNA Method

**FIGURE 2:**
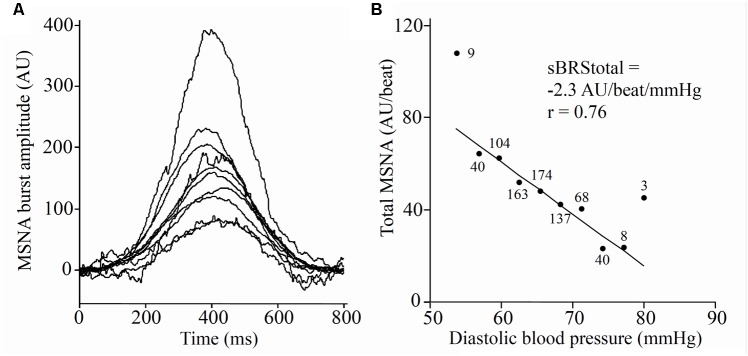
Sympathetic baroreflex assessment in a 21-year-old male using the segregated signal averaging approach. **(A)** MSNA bursts are normalized to the burst with the largest amplitude and entered into diastolic pressure bins of 3 mmHg. Each line represents the mean MSNA burst amplitude for a different bin, with the largest representing the lowest diastolic pressure bin. **(B)** Total MSNA per beat is determined for each bin and plotted against diastolic pressure (Taylor et al., 2015). Number of cardiac cycles per bin indicated next to each data point.

The largest MSNA burst for each 5-min period and the 2-min period was assigned a value of 1000 and the remaining MSNA bursts during each period were normalized against this (Halliwill, 2000). The relationship between diastolic pressure and total MSNA was assessed using 3 mmHg bins. Total integrated MSNA was determined for each bin using a segregated signal averaging approach (Halliwill, 2000) and expressed as arbitrary units (AU) per beat. **Figure 2A** shows the mean MSNA burst amplitude for each bin. The lower diastolic pressure bins have a higher mean amplitude, and the higher diastolic pressure bins have a lower mean amplitude. Linear regression was used to determine the relationship between total MSNA and diastolic pressure as shown in **Figure 2B**, with the application of the weighting procedure described above to account for the number of cardiac cycles per bin. These baroreflex values will be referred to as ‘sympathetic BRS_total_’ to differentiate them from the MSNA burst incidence method for assessing sympathetic BRS.

The diastolic pressure range was determined for the first 5-min and the 2-min recordings for each participant by calculating the difference between the highest and lowest diastolic pressure bins from the BRS analysis. Diastolic pressure ranges in males and females were compared between 5- and 2-min recording periods to determine whether the duration of the recording affects the range of diastolic pressures involved in the baroreflex assessment.

### Statistical Analysis

The following statistical analysis was performed separately for males and females. Comparisons were made between the two 5-min periods to examine the stability of BRS within a single recording session. Intra-class correlations, ordinary least products regression (OLP) and Bland–Altman plots were employed to compare the two 5-min periods to determine if sympathetic BRS is stable; to compare the first 5-min period with the 2-min recording to examine the effect of recording duration; and finally, in a subgroup of participants, the first 5-min recording was compared with another 5-min recording measured on a separate day to examine between day repeatability.

#### Intra-Class Correlations

These were performed for each comparison to test for repeatability in BRS values. Intra-class correlations of <0.5, 0.5–0.74, 0.75–0.89, and >0.90 are indicative of poor, moderate, good, and excellent repeatability, respectively (Koo and Li, 2016).

#### Ordinary Least Products Regression

The goal of repeatability studies is not to find similarities between two recording periods but to determine whether any systematic differences or bias exists (Ludbrook, 1997). Therefore, OLP regression analyses were performed to determine whether there is any error or bias between the two BRS values. OLP is recommended over the more common ordinary least squares (OLS) regression as the recordings being compared both have some form of error (Ludbrook, 1997). All calculations were performed using techniques described by Ludbrook (1997). The 95% confidence intervals (CI) of the intercept (α′) and slope (*b*′) of the relationship will allow the presence of any fixed (where the intercept differs from zero) or proportional (where the slope of the regression differs from unity, i.e., 1) bias to be determined (Ludbrook, 1997). If the 95% CI for the intercept contains ‘0’ and the 95% CI for the slope contains ‘1’ then no proportional or fixed bias exists.

#### Bland–Altman Plots

This analysis allows us to plot the difference between the two BRS values against the mean value of the two BRS values (Altman and Bland, 1983; Bland and Altman, 1986; Ludbrook, 1997). Bland–Altman plots also provided a visual representation of how the data is spread (Altman and Bland, 1983; Bland and Altman, 1986; Ludbrook, 1997). A secondary examination of bias/error was performed on the Bland–Altman test. Proportional bias was determined using OLS regression of the mean against the difference of the two BRS values for BRS_inc_ and BRS_total_. If the slope of the OLS regression was significantly different from ‘0,’ then proportional bias exists. A one-sample *t*-test of the mean difference was tested against a value of ‘0’ to determine whether fixed bias exists. Fixed bias was evident if the one-sample *t*-test was significantly different from 0.

#### Comparing Diastolic Pressure Ranges

Students paired *t*-tests were performed to compare diastolic blood pressure ranges between the first 5-min recording and the 2-min recording (taken from the second 5-min period). Linear regression analysis was performed between diastolic pressure ranges in the 5- and 2-min recordings with the corresponding BRS values to examine the influence of diastolic pressure ranges on BRS values.

Statistical analyses were performed using Prism v6.00 for Windows (GraphPad Software, San Diego, CA, United States). Acceptance levels for BRS slopes were *r* ≥ 0.5. For all statistical tests, a probability level of *P* ≤ 0.05 was regarded as significant. All values are expressed as mean ± standard deviation (SD).

## Results

### Sex Differences in Cardiovascular Variables

When comparing males and females using the first 5-min rest period, males had higher MSNA burst incidence and MSNA burst frequency. There was no significant difference in systolic pressure, diastolic pressure, mean arterial pressure or heart rate between males and females (**Table 1**). However, females had significantly greater sympathetic BRS when expressed using both MSNA burst incidence (*p* = 0.001) and MSNA total activity (*p* = 0.03). There was no significant difference in the number of cardiac cycles between the first and second 5-min periods in males (331 ± 33 vs. 331 ± 33 cardiac cycles; *p* = 0.67) and females (344 ± 38 vs. 342 ± 39 cardiac cycles; *p* = 0.24).

**Table 1:** Mean values for systolic BP, diastolic BP, MAP, HR, and MSNA during 5- and 2-min recording periods in males (*n* = 47) and females (*n* = 35).

Cardiovascular variable	Males 5-min	Males 2-min	Females 5-min	Females 2-min
	mean ± *SD*	mean ± *SD*	mean ± *SD*	mean ± *SD*
Systolic BP (mmHg)	128 ± 18	128 ± 19	125 ± 19	124 ± 19
Diastolic BP (mmHg)	65 ± 12	65 ± 11	64 ± 12	63 ± 12
MAP (mmHg)	83 ± 12	83 ± 12	83 ± 13	82 ± 13
Heart rate (bpm)	66 ± 8	66 ± 8	69 ± 7	69 ± 8
Burst incidence (bursts/100heartbeats)	45 ± 13	52 ± 14	38 ± 11	37 ± 11*
Burst frequency (bursts/minute)	29 ± 7	33 ± 8	25 ± 6	24 ± 6*

*^∗^Indicates a significant difference (p < 0.05) between males and females.*

### Intra-Class Correlations

Acceptable sympathetic BRS_inc_ values (*r* ≥ 0.5) were obtained for both 5-min periods in 82 participants (47 males and 35 females). The intra-class correlation coefficient suggests moderate stability in sympathetic BRS_inc_ between the first and second 5-min periods in males (-3.2 ± 1.4 vs. -3.2 ± 1.5 bursts/100 HB/mmHg; *r* = 0.63) and females (-4.4 ± 1.8 vs. 3.8 ± 1.6 bursts/100 HB/mmHg; *r* = 0.61). Acceptable sympathetic BRS_total_ values were obtained for both 5-min periods in 42 participants (26 males and 16 females). There was a strong correlation in sympathetic BRS_total_ between the first and second 5-min periods in males (-6.9 ± 3.6 vs. -6.5 ± 3.8 AU/beat/mmHg; *r* = 0.78) but a poor repeatability in females (-9.4 ± 3.8 vs. -9.1 ± 5.2 AU/beat/mmHg; *r* = 0.47).

Acceptable sympathetic BRS_inc_ values were obtained in 76 participants (45 males, 31 females) when comparing the 5 and 2-min recordings. The intra-class correlation coefficient indicated poor repeatability in BRS_inc_ between the 5- and 2-min recording periods in males (-3.2 ± 1.3 vs. -3.3 ± 1.4 bursts/100 HB/mmHg; *r* = -0.01) and females (-4.4 ± 1.9 vs. -4.5 ± 2.3 bursts/100 HB/mmHg; *r* = 0.46). Acceptable BRS_total_ values were acquired in 44 participants (30 males, 14 females) when comparing 5- and 2-min recording periods. There was a moderate intra-class correlation for sympathetic BRS_total_ between the first 5- and 2-min recording periods in males (-6.6 ± 3.4 vs. -8.8 ± 4.2 AU/beat/mmHg; *r* = 0.70) but a poor correlation in females (-9.2 ± 4.0 vs. -13.6 ± 8.4 AU/beat/mmHg; *r* = 0.39).

Of the 13 participants who returned on a separate day for a second experimental session, acceptable sympathetic BRS_inc_ values were obtained for 11 participants (2 female). The intra-class correlation coefficient suggests a moderate correlation between the two 5-min periods (-3.28 ± 1.0 bursts/100 HB/mmHg vs. -2.79 ± 1.84 bursts/100 HB/mmHg, *r* = 0.63). Acceptable BRS_total_ values were acquired in eight participants with a moderate intra-class correlation (-4.60 ± 3.2 AU/beat/mmHg vs. -8.28 ± 5.7 AU/beat/mmHg, *r* = 0.68).

### Ordinary Least Products Regression Analysis

**FIGURE 3:**
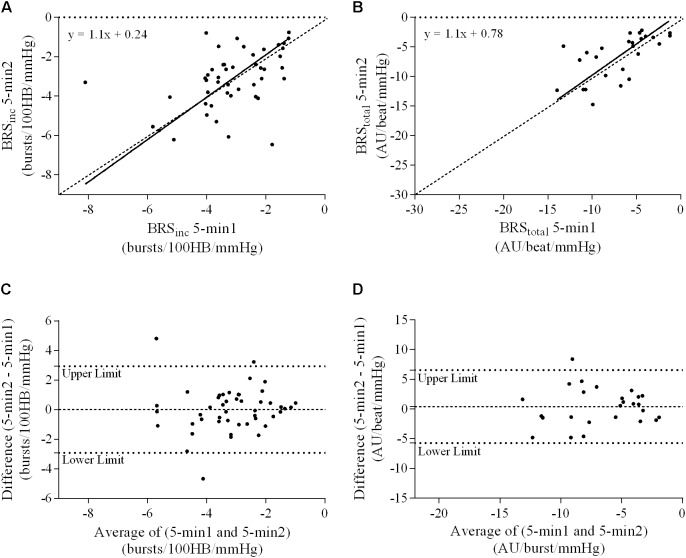
Sympathetic BRS quantified using the first 5-min period (5-min1) and second 5-min period (5-min2) in males. Ordinary least products (OLP) regression analysis using **(A)** BRS_inc_ and **(B)** BRS_total_ and Bland–Altman plots using **(C)** BRS_inc_ and **(D)** BRS_total_. The solid and dashed lines in the OLP plots represent the line of regression and line of unity, respectively. The dashed and dotted lines in the Bland–Altman plots represent mean difference and 95% limits of agreement, respectively.

**FIGURE 4:**
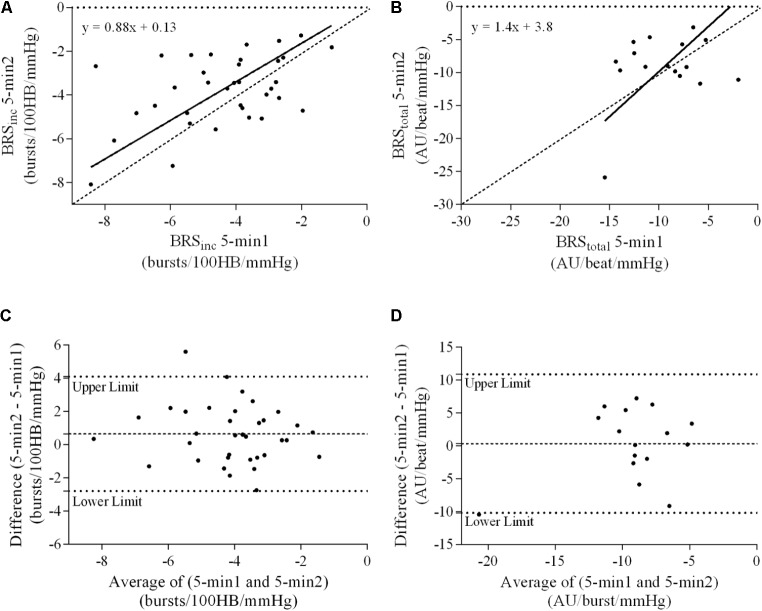
Sympathetic BRS quantified using the first 5-min period (5-min1) and second 5-min period (5-min2) in females. Ordinary least products (OLP) regression analysis using **(A)** BRS_inc_ and **(B)** BRS_total_ and Bland–Altman plots using **(C)** BRS_inc_ and **(D)** BRS_total_. The solid and dashed lines in the OLP plots represent the line of regression and line of unity, respectively. The dashed and dotted lines in the Bland–Altman plots represent mean difference and 95% limits of agreement, respectively.

**FIGURE 5:**
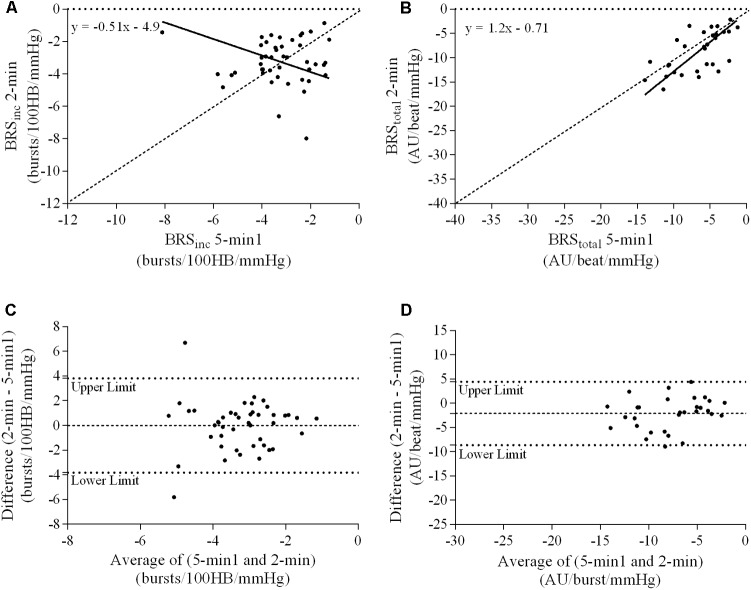
Sympathetic BRS quantified using the first 5-min period (5-min1) and 2-min recording period in males. Ordinary least products (OLP) regression analysis using **(A)** BRS_inc_ and **(B)** BRS_total_ and Bland–Altman plots using **(C)** BRS_inc_ and **(D)** BRS_total_. The solid and dashed lines in the OLP plots represent the line of regression and line of unity, respectively. The dashed and dotted lines in the Bland–Altman plots represent mean difference and 95% limits of agreement, respectively.

**FIGURE 6:**
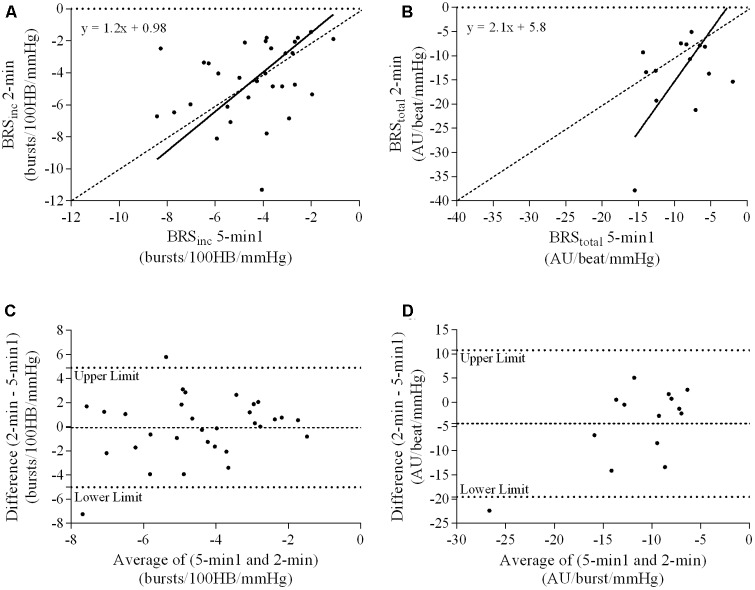
Sympathetic BRS quantified using the first 5-min period (5-min1) and 2-min recording period in females. Ordinary least products (OLP) regression analysis using **(A)** BRS_inc_ and **(B)** BRS_total_ and Bland–Altman plots using **(C)** BRS_inc_ and **(D)** BRS_total_. The solid and dashed lines in the OLP plots represent the line of regression and line of unity, respectively. The dashed and dotted lines in the Bland–Altman plots represent mean difference and 95% limits of agreement, respectively.

**FIGURE 7:**
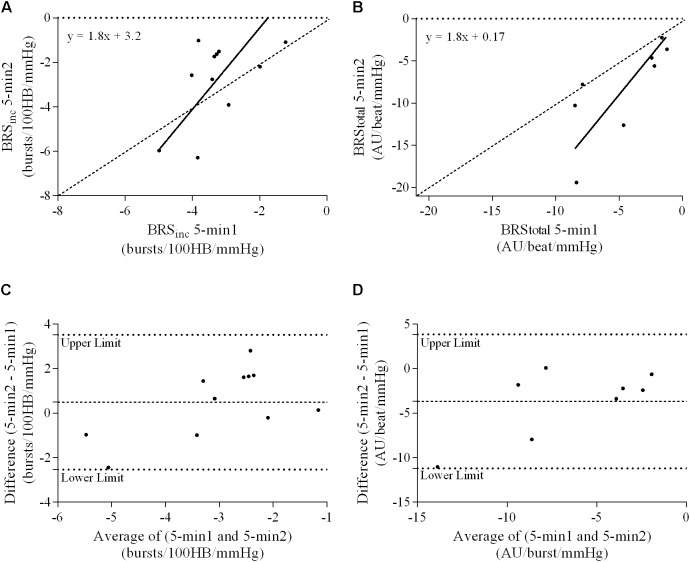
Sympathetic BRS quantified using two 5-min periods recorded on separate days. Ordinary least products (OLP) regression analysis using **(A)** BRS_inc_ and **(B)** BRS_total_ and Bland–Altman plots using **(C)** BRS_inc_ and **(D)** BRS_total_. The solid and dashed lines in the OLP plots represent the line of regression and line of unity, respectively. The dashed and dotted lines in the Bland–Altman plots represent mean difference and 95% limits of agreement, respectively.

A summary of OLP regression analysis for BRS_inc_ and BRS_total_ is provided in **Table 2**. The results of the OLP analysis indicate that there was no evidence of fixed or proportional bias between the two baroreflex slopes for both BRS_inc_ and BRS_total_ when comparing the two 5-min recording periods, between the first 5- and 2-min periods in both males and females, and also in the subgroup of participants who returned on a separate day. This means that (1) one recording period did not have values that were different from the second by a constant amount over the total range of BRS values (fixed bias); and (2) one recording did not give values that were different from those from the second by an amount that was proportional to the level of the BRS variable (proportional bias) (Ludbrook, 1997; Kimmerly et al., 2004). **Figures 3A,B** illustrate the comparisons between the first and second 5-min recording periods for BRS_inc_ and BRS_total_ in males, and **Figures 4A,B** illustrates these comparisons in females. The comparison between the 5- and 2-min recording periods in males is illustrated in **Figures 5A,B**, and in females in **Figures 6A,B**. The between-session comparisons are illustrated in **Figures 7A,B**.

**Table 2:** Ordinary least products regression of sympathetic baroreflex sensitivity.

Variable	ICC	α′	95%CI for α′	*b*′	95% CI for *b*′	Fixed bias	Proportional bias
**MALES**
BRS_inc_ 5 min1 vs. 5 min2 (*n* = 47)	0.63	0.24	-1.93, 2.42	1.08	0.44, 1.71	NO	NO
BRS_total_ 5 min1 vs. 5 min2 (*n* = 26)	0.78	0.78	-3.42, 4.98	1.06	0.51, 1.6	NO	NO
BRS_inc_ 5 min1 vs. 2 min (*n* = 45)	-0.01	-4.9	-85.53, 75.73	-0.54	-23.51, 22.49	NO	NO
BRS_total_ 5 min1 vs. 2 min (*n* = 30)	0.70	-0.71	-5.02, 3.6	1.21	-0.63, 1.79	NO	NO
**FEMALES**					
BRS_inc_ 5 min1 vs. 5 min2 (*n* = 35)	0.61	-0.14	-2.66, 2.91	0.88	0.30, 1.47	NO	NO
BRS_total_ 5 min1 vs. 5 min2 (*n* = 16)	0.47	3.8	-20.97, 28.56	1.37	-1.08, 3.81	NO	NO
BRS_inc_ 5 min1 vs. 2 min (*n* = 31)	0.46	0.98	-6.2, 8.2	1.23	-0.26, 2.7	NO	NO
BRS_total_ 5 min1 vs. 2 min (*n* = 14)	0.39	5.78	-24.68, 36.24	2.11	-0.95, 5.17	NO	NO

*ICC, intra-class correlation coefficient; α′, b′ coefficients of ordinary least products regression model (Y) = a′+b′(X); a′, oxy-intercept; b′, slope, fixed bias, if 95%CI for a′ does not include 0, proportional bias, if 95%CI for c′ does not include 1.*

### Bland–Altman Analysis

Results of the Bland–Altman plots are detailed in **Table 3**. In males, the mean difference in BRS_inc_ between the two 5-min periods was 0.07 ± 1.5 bursts/100 HB/mmHg, and for BRS_total_ was -0.40 ± 3.1 AU/beat/mmHg. For females, the mean difference in BRS_inc_ between the two 5-min periods was 0.65 ± 1.8 bursts/100 HB/mmHg, and for BRS_total_ was 0.35 AU/beat/mmHg. In males, the mean difference in BRS_inc_ between the 5- and 2-min periods was -0.01 ± 1.9 bursts/100 HB/mmHg, and for BRS_total_ was -2.13 ± 3.3 AU/mmHg. For females, the mean difference in BRS_inc_ between the 5- and 2-min periods was -0.05 ± 2.5 bursts/100 HB/mmHg, and for BRS_total_ was -4.38 ± 7.7 AU/beat/mmHg. When comparing the BRS values between the two 5-min recording periods, there was no evidence of proportional bias in BRS_inc_ or BRS_total_, as illustrated in **Figures 3C,D** (males) and **Figures 4C,D** (females). However, there was evidence of fixed bias for BRS_inc_ in females with higher BRS values for the first 5-min recording. When comparing the BRS values from the first 5-min with the 2-min recording period, there was no proportional or fixed bias when BRS was quantified using MSNA burst incidence. However, there was evidence of fixed bias for BRS_total_ in males, with higher BRS values acquired from the 2-min recording, and proportional bias for BRS_total_ in females. When comparing the BRS values that were acquired from two experimental sessions, there was evidence of proportional bias in BRS_inc_ and fixed bias in BRS_total_. These comparisons are illustrated in **Figures 5C,D** for males, **Figures 6C,D** for females, and **Figures 7C,D** in the subgroup of participants.

**Table 3:** Proportional and fixed bias outcomes for sympathetic baroreflex sensitivity from Bland–Altman.

Variable	*R*	*B*	P(OLS)	Proportional bias	Mean difference ±*SD*	Mean difference 95%CI	*P*(*t*-test)	Fixed bias
**MALES**					
BRS_ inc_ 5 min1 vs. 5min2	0.08	0.09	0.61	NO	0.07 ± 1.5	-0.43, 0.44	0.98	NO
BRS_ total_ 5 min1 vs. 5min2	0.07	0.07	0.73	NO	-0.40 ± 3.1	-0.87, 1.66	0.52	NO
BRS_ inc_ 5 min1 vs. 2min	0.04	0.08	0.80	NO	-0.01 ± 1.9	-0.59, 0.58	0.98	NO
BRS_ total_ 5 min1 vs. 2min	0.24	0.24	0.19	NO	-2.13 ± 3.3	-3.37, -0.88	0.002	YES
**FEMALES**					
BRS_inc_ 5 min1 vs. 5 min2	0.14	0.17	0.42	NO	0.65 ± 1.8	0.04, 1.25	0.04	YES
BRS_total_ 5 min1 vs. 5 min2	0.32	0.47	0.23	NO	0.35 ± 5.4	-2.5, 3.2	0.80	NO
BRS_inc_ 5 min1 vs. 2 min	0.22	0.33	0.23	NO	-0.05 ± 2.5	-0.98, 0.87	0.90	NO
BRS_total_ 5 min1 vs. 2 min	0.66	0.97	0.009	YES	-4.38 ± 7.7	-8.85, 0.09	0.05	NO

*r, product-moment correlation for Bland–Altman method of differences plots; b, ordinary least squares slope of Bland–Altman method of differences, P(OLS), the p-value for the ordinary least square slope (versus 0); CI, confidence interval; p (t-test), the p-values for the one sample t-test on the mean differences (versus 0).*

### Diastolic Pressure Ranges

In males, the diastolic pressure range was significantly greater in the 5-min recording period (21 ± 6 mmHg) when compared with the 2-min recording period (16 ± 6 mmHg, *P* < 0.01). Similarly, in females, the diastolic pressure range was significantly greater in the 5-min recording period (17 ± 6 mmHg) compared with the 2-min recording period (14 ± 6 mmHg, *P* = 0.01). In males, the diastolic pressure range was inversely related to the BRS; the greater the diastolic pressure range, the lower the individual’s BRS. This was demonstrated for both the 5- and 2-min recordings when BRS was expressed as BRS_inc_ (5-min *r* = 0.44, *p* < 0.01; 2-min *r* = 0.47, *p* < 0.01) and BRS_total_ (5-min *r* = 0.68, *p* < 0.01; 2-min *r* = 0.54, *p* < 0.01). In females, the diastolic pressure range was also inversely related to BRS in the 5-min period when expressed as BRS_inc_ (*r* = 0.55, *p* < 0.01) but not BRS_total_ (*r* = 0.03, *p* = 0.92). Similarly when quantifying BRS using 2-min recording, diastolic pressure range was inversely related with BRS_inc_ (*r* = 0.57, *p* < 0.01) and trended toward significance with BRS_total_ (*r* = 0.45, *p* = 0.10).

## Discussion

When performing baroreflex assessments the results are only meaningful if we understand the stability of baroreflex sensitivity and if the analytical technique used to quantify spontaneous sympathetic BRS is repeatable. This is the first study to employ appropriate statistical tests to examine the stability and repeatability of spontaneous sympathetic BRS techniques. The main findings of this study are (i) both BRS_inc_ and BRS_total_ are moderately stable within a recording session when using periods of the same duration, with no evidence of fixed or proportional bias in males, but proportional bias in females; (ii) there is generally poor repeatability when comparing recording periods of differing length (5-min vs. 2-min) with evidence of fixed and proportional bias in BRS_total_ values; and (iii) when measured on separate days, BRS is moderately repeatable with evidence of proportional bias in BRS_inc_ and fixed bias in BRS_total_.

### The Stability and Repeatability of Baroreflex Sensitivity

Sympathetic baroreflex sensitivity is reportedly lower in certain populations, such as those with hypertension (Greaney et al., 2017), heart failure (Ferguson et al., 1992), and obstructive sleep apnoea (Carlson et al., 1996). However, evidence on the repeatability of sympathetic BRS is limited. The quantification of BRS has predominantly been performed using the cardiac arm of the baroreflex as it is easily obtained non-invasively via ECG and beat-to-beat measurements of systolic blood pressure (Parati et al., 1988). Previous studies have shown cardiac BRS, quantified using analytical techniques such as the sequence method and spectral analysis, to be repeatable within the same session and over a period of weeks to months (Dawson et al., 1997; Herpin and Ragot, 1997; Davies et al., 1999; Johnson et al., 2006). The quantification of sympathetic BRS involves techniques similar to that of cardiac BRS. However, due to the invasive nature of microneurography, less research has been conducted on sympathetic BRS. These experiments are more technically demanding and require significant expertise. Moreover, the amplitude of a burst of MSNA depends on the proximity of the microelectrode tip to the active sympathetic axons, so absolute amplitudes cannot be directly compared across experimental sessions, though relative amplitudes can. There has been only one other study where the repeatability of sympathetic BRS was examined. In this study, Kienbaum et al. (2001) reported spontaneous sympathetic BRS to be repeatable within the same session in healthy adults. However, the only statistical analysis employed was the Student’s paired *t*-test. It is unlikely that this approach is sensitive enough to reveal systematic differences in BRS values obtained from the two recordings. Nevertheless, it does not provide information on the correlation between repeat tests and, in particular, whether there are large intra-individual differences. In the current study, there were moderate intra-class correlations for BRS_inc_ and BRS_total_ between the two 5-min periods in both males and females. There was also moderate repeatability in these variables when assessed on different days. The fact that these relationships are not stronger may reflect the dynamic nature of the baroreflex, indicating that baroreflex sensitivity is not fixed, even under resting conditions. By evaluating sympathetic BRS from two segments in the same recording period we can gain an appreciation of the stability of BRS. The data suggest that the baroreflex may be constantly adapting and reacting to small changes in the internal environment despite no changes in the experimental conditions. Furthermore, what constitutes a meaningful difference in sympathetic BRS has yet to be established. In previous studies statistically significant changes in sympathetic BRS have been reported in the range of 1.5–2.91 bursts/100 HB/mmHg following various interventions, such as heat stress (Keller et al., 2006), renal denervation (Hart et al., 2013) and insulin (Young et al., 2010), as well as between phases of the menstrual cycle (Carter et al., 2009). In the current study the mean difference in BRS_inc_ between the two 5-min recording periods was 0.07 ± 1.5 bursts/100 HB/mmHg for males, and 0.65 ± 1.8 bursts/100 HB/mmHg for females. Based on the previous literature, the mean difference may be deemed too small to be meaningful, thus supporting the view that BRS is relatively stable at rest. However, the variation suggests that for some individuals the differences were comparable to the changes in sympathetic BRS observed in intervention studies. Nevertheless, statically significant changes do not necessarily reflect meaningful ones and there is currently no consensus on what constitutes a low sympathetic BRS value or a meaningful change. Further research is needed to establish clinical thresholds associated with elevated cardiovascular risk and thus meaningful changes in sympathetic BRS following interventions. Further research is also required to determine the normal ranges of within and between-subject variability in sympathetic BRS in healthy populations.

Although intra-class correlations provides some evidence of repeatability in measures of BRS, this approach does not offer any indication of the error or bias in the data. To explore the systematic differences between repeat baroreflex assessments, we performed OLP regression and Bland–Altman analysis and found no fixed or proportional bias when comparing BRS values using two consecutive 5-min recordings in males. This suggests that spontaneous sympathetic BRS analysis is not susceptible to bias within the same session. However, there was evidence of proportional bias in females when BRS was quantified using MSNA burst incidence. Moreover, when comparing between two recording periods of different lengths, fixed and proportional bias was evident when BRS was quantified using total MSNA. It is therefore recommended that studies involving repeat measurements of BRS include intervals of the same duration. Previous studies have reported spontaneous sympathetic BRS using intervals of 1-min (Ichinose et al., 2006), 4-min (Ichinose et al., 2004b; Ogoh et al., 2007), 5-min (Kienbaum et al., 2001; Ichinose et al., 2004a; Keller et al., 2006; Hart et al., 2010; Hinojosa-Laborde et al., 2014) and 10-min (Hissen et al., 2015, 2017; Taylor et al., 2015). The current study suggests that care should be taken when comparing BRS values between studies when different time periods have been used.

### Sex Differences in Sympathetic Baroreflex Sensitivity

Previous research indicates that several aspects of cardiovascular control differ between males and females. For instance, studies have shown that females have lower blood pressure, cardiac output and vascular transduction when compared with males (Joyner et al., 2015; Briant et al., 2016). Furthermore, resting MSNA in males is inversely related to cardiac output (Hart et al., 2009), which may explain how young healthy males can have similar resting blood pressure levels despite differing levels of MSNA. Conversely, this relationship is not apparent in premenopausal females (Hart et al., 2011a). Previous evidence suggests this may be due to enhanced β-adrenergic sensitivity in females, which therefore balances the vasoconstrictor effects of sympathetic outflow (Kneale et al., 2000; Hart et al., 2011a). In the current study, baroreflex control of MSNA, quantified using both MSNA burst incidence and total activity was greater in females, as has been reported previously (Hogarth et al., 2007). However, others have reported no differences between males and females (Tank et al., 2005; Hart et al., 2011b). Although there is evidence to suggest females have higher sympathetic BRS, it may not necessarily demonstrate enhanced baroreflex buffering of arterial pressure as β-adrenergic receptors counteract the vasoconstrictor nature of MSNA (Hart et al., 2011a).

### Indices of Sympathetic Baroreflex Sensitivity

In this study, spontaneous sympathetic BRS was quantified using both MSNA burst incidence and total MSNA and thus can contribute to debates around the most appropriate characteristics of MSNA for baroreflex analysis. When using a 5-min recording, there was a higher success rate for acquiring an acceptable baroreflex slope (*r* > 0.5) using MSNA burst incidence (82 out of 84) than when using total MSNA (42 out of 84). Kienbaum et al. (2001) previously showed that the quantification of spontaneous BRS was more successful when using MSNA burst incidence (referred to as threshold analysis) than MSNA burst amplitude which gave rise to the hypothesis that there are two central nervous system pathways of MSNA. The results indicated that the baroreflex modulation of MSNA is more closely related to the occurrence of MSNA bursts than the strength of MSNA bursts. This may be due to non-baroreflex inputs such as respiration dominating over the baroreflex to determine the size of a sympathetic burst (Hart et al., 2010). However, previous studies suggest that the amplitude of MSNA bursts is an important and influential factor in the changes in arterial pressure (Vianna et al., 2012; Fairfax et al., 2013). While it may be argued that methods involving total integrated MSNA provide a more comprehensive assessment of baroreflex modulation of MSNA, the current study supports previous evidence that the MSNA burst incidence approach provides the most robust BRS slopes (Kienbaum et al., 2001; Taylor et al., 2015).

The lack of significant BRS_inc_ and BRS_total_ slopes in some individuals may be due to an insufficient blood pressure range within the 2-min recording period. A 2-min time interval may not contain sufficient spontaneous fluctuations in diastolic pressure and the corresponding MSNA. Longer recording periods may allow enough time for a larger diastolic blood pressure range and therefore explain why in the current study there was a higher success rate of acquiring a sympathetic BRS slope with a linear relationship of *r* > 0.5 using the 5-min period (82/84 for BRS_inc_ and 42/84 for BRS_total_). In this study, the 5-min recording period was associated with a significantly larger diastolic blood pressure range when compared with the 2-min recording period in both males and females. When comparing the 5-min recording with the 2-min recording, intra-class correlation coefficients indicated poor to moderate repeatability. The Bland–Altman analysis also revealed fixed bias for BRS_total_ in males and proportional bias for BRS_total_ in females. The results suggest that, in males, BRS_total_ values quantified using the 2-min recording period were higher by a constant amount when compared with values from the 5-min period. In females, BRS_total_ values from the 2-min recording increased in proportion to the BRS values in the 5-min recording. Regression analyses revealed that larger diastolic blood pressure ranges are associated with lower BRS values. This suggests that the use of very short recordings, such as 2 min, is associated with limited pressure ranges and artificially high BRS values. The results also highlight the importance of comparing BRS values using recording periods that are of the same duration to ensure that the intervention, stressor or change in environment is the actual cause of the shift in BRS.

### Methodological Considerations

To date, the quantification of sympathetic BRS has been performed predominantly through spontaneous techniques (Ichinose et al., 2008; Hart et al., 2010, 2011b; Hissen et al., 2015). However, methods involving active perturbation of blood pressure, such as the modified Oxford method, have also been applied. This technique involves bolus injections of sodium nitroprusside and phenylephrine to drive decreases and increases in blood pressure, respectively (Ebert and Cowley, 1992). This is advantageous as it partially opens the closed-loop system of the baroreflex, allowing estimates of the ratio of the inputs and outputs of the baroreflex to be observed (Lipman et al., 2003; Diaz and Taylor, 2006). Although the modified Oxford method is regarded as the gold standard for quantifying cardiac BRS, its use in determining sympathetic BRS can raise some technical issues (Dutoit et al., 2010; Taylor et al., 2014). For instance, when assessing cardiac BRS, there is an R-R interval for every cardiac cycle plotted against systolic pressure. However, when determining sympathetic BRS, not every cardiac cycle is associated with an MSNA burst, and it is not uncommon for the administration of phenylephrine to cause rapid and severe inhibition of MSNA bursts, making it difficult to plot MSNA/diastolic pressure relationships (Taylor et al., 2014). Spontaneous methods are therefore often preferred for the sympathetic baroreflex, but future research on the repeatability of approaches involving active perturbations is important as these techniques provide more rapid changes in pressure, typically over a broader range.

When comparing measurements of MSNA and sympathetic BRS between segments of data, it is important to take heart rate into account. The more cardiac cycles within the segment, the greater the probability of a burst of MSNA and thus the more data points available for assessing sympathetic BRS. In this study there were no significant differences in the number of cardiac cycles in segments of the same duration, largely due to all comparisons being made at rest. However, in studies where sympathetic BRS is compared between different conditions, such as rest and mental stress, differences in heart rate may be present. In these cases, it may be more appropriate to control for the number of cardiac cycles per segment, rather than segment duration.

## Conclusion

This study demonstrates, for the first time, that sympathetic BRS is moderately stable within a single recording session. When quantified using both MSNA burst incidence and total MSNA there is no fixed or proportional bias present in males. There is, however, proportional bias for BRS_inc_ in females. When data segments of different durations are used, intra-class correlations generally indicate poor repeatability with both fixed and proportional bias. Recordings of a shorter duration were associated with small diastolic blood pressure ranges and artificially elevated BRS values. When sessions are performed on separate days the analytical techniques used to quantify spontaneous sympathetic BRS are associated with moderate repeatability in healthy young males and females. Results from this study indicate that measures of spontaneous sympathetic BRS are moderately repeatable but only when the duration of recording periods are the same. Future research is required to examine the interactions between sex and aging, and to establish clinical thresholds in sympathetic BRS.

## Author Contributions

Experiments were performed in the School of Medicine (Western Sydney University). All authors were involved in the design of the experiments and/or data acquisition and analysis of the data, as well as the writing or editing of this manuscript. All authors approved the final version of this manuscript and agreed to be accountable for all aspects of the work.

## Conflict of Interest Statement

The authors declare that the research was conducted in the absence of any commercial or financial relationships that could be construed as a potential conflict of interest.
